# Analysis of Telestroke Usage in Rural Critical Access Emergency Departments

**DOI:** 10.1089/tmj.2022.0408

**Published:** 2023-12-08

**Authors:** Priya Arumuganathan, Amelia K. Adcock, Cristal Espinosa, Scott Findley

**Affiliations:** ^1^ Department of Emergency Medicine, School of Medicine, West Virginia University, Morgantown, West Virginia, USA.; ^2^ Department of Neurology, School of Medicine, West Virginia University, Morgantown, West Virginia, USA.; ^3^ Department of Neurology, Mayo Clinic, Phoenix, Arizona, USA.; ^4^ Department of Pediatric Emergency Medicine, West Virginia University, Morgantown, West Virginia, USA.

**Keywords:** *telemedicine*, *telestroke*, *telehealth*, *teleneurology*, *emergency medicine*

## Abstract

**Introduction:**

*Telestroke is an effective strategy to increase appropriate stroke treatments among patients in resource-limited environments. Despite the well-documented benefits of telestroke, there is limited literature regarding its utilization. The purposes of this study are: (1) determine the percentage of potential stroke patients who generate a telestroke consult in rural critical access hospitals (CAHs) and (2) validate an electronic medical record (EMR)-derived report as a stroke screen.*

**Methods:**

*This retrospective chart review analyzed patients presenting between September 1, 2020 and February 1, 2021 to three CAHs. Visits with triage complaints suggesting acute ischemic stroke (AIS)/transient ischemic attack (TIA) were pooled for analysis using an EMR-derived report. Patients with confirmed AIS/TIA at discharge over this period were used to validate the EMR tool.*

**Results:**

*The EMR report pooled 252 possible AIS/TIA visits out of 12,685 emergency department visits for analysis. It had a specificity of 98.78% and sensitivity of 58.06%. Of the 252 visits, 12.7% met telestroke criteria and 38.89% received telestroke evaluation. Among these, a definite diagnosis of AIS/TIA was made in 92.86%. Of the remaining population who met criteria but didn't undergo consultation, 61.11% were diagnosed with AIS/TIA at discharge.*

**Conclusion:**

*This study provides novel characterization of stroke presentations and telestroke in rural CAHs. The EMR-derived report is a reasonable tool to concentrate potential AIS/TIA cases for review and resource allocation but is not sensitive enough to detect stroke as a stand-alone tool. The majority (56%) of eligible patients did not undergo telestroke consultation. Future studies are critical to further understand reasons contributing to this.*

## Introduction

Stroke remains one of the leading causes of morbidity and mortality worldwide, especially in rural and remote areas.^1^ The majority of stroke cases are ischemic, which is amenable to evidence-based interventions such as endovascular thrombectomy (EVT) and intravenous thrombolysis (IVT).^2^ However, these interventions are time-sensitive and require familiarity with stroke guidelines, as well as access to adequately-equipped centers. At facilities where there are no on-site stroke physicians, telestroke is an established tool to aid in clarifying a stroke/transient ischemic attack (TIA) diagnosis and ensuring access to the most appropriate treatments for eligible patients. In hub-and-spoke models, care is provided using a networking stroke physician at a distant site, known as the “hub,” with the originating facility, known as the “spoke.”^3^

The diagnostic accuracy of telestroke has been demonstrated to be comparable to in-person neurology consultation, and it is one of the most effective strategies to increase appropriate acute ischemic stroke (AIS) treatments in resource limited environments.^4^ The benefits of telestroke have been demonstrated by improvements in time-to-consult and door-to-needle times.^5^ In our own rural region, the telestroke program increased IVT usage by 73% within the network's facilities, while rates of hemorrhagic conversion remained at 1%.^6^

Despite the well-known benefits of telestroke services, there are very limited data regarding telestroke utilization rates, definitions of what constitutes optimal utilization, and barriers to its utilization. The objectives of our study, therefore, are: (1) report the frequency and characteristics of telestroke consults generated at three rural critical access hospitals (CAHs) and (2) examine the reliability of an electronic medical record (EMR)-derived report as a screening tool to identify potential AIS/TIA cases.

## Methods

This study was approved by the West Virginia University (WVU) Institutional Review Board. In compliance with national legislation and institutional requirements, written informed consent was waived. This retrospective chart review analyzed three CAHs participating in the WVU telestroke network. All patients presenting to the emergency department (ED) between September 1, 2020 and February 1, 2021 with triage complaints associated with AIS or TIA were pooled for analysis (*n* = 252) using an EMR-derived report (*Table 1*). The WVU Telestroke Program's activation criteria (shown in *Table 2*) were used to identify patients most likely to benefit from telestroke consultation. Consistent application of the criteria across reviewers (A.K.A., C.E., P.A.) was validated using a cross-check among a random sample. The frequency of telestroke activation, number of patients meeting telestroke activation criteria, and rates of stroke in both the telestroke and nontelestroke patients were recorded. Individual charts were also reviewed to determine reasons that providers did not utilize the telestroke service. Sensitivity and specificity analysis was used to determine the viability of the EMR Report as a tool for identifying cases of AIS/TIA. Deidentified patient data were recorded on Microsoft Excel and analyzed with Omni Calculator.^7^ Validity was assessed by cross-referencing the EMR report across all patients with a discharge diagnosis of acute AIS/TIA during the same time period.

**Table 1.** Triage Complaints Captured in the Electronic Medical Record Report

TRIAGE COMPLAINT
• Confusion
• Dizziness
• Extremity weakness
• Facial droop
• Facial numbness/tingling
• Headache
• Muscle weakness
• Neurological deficit
• Numbness
• Slurred speech
• Stroke alert
• Stroke—S&S
• Transient ischemic attack
• Vision change
• Vision loss
• Visual field change

**Table 2.** Telestroke Activation Criteria

A PROVIDER MAY CHOOSE TO ACTIVATE TELESTROKE SERVICES IF THERE IS INDICATION THAT THE PATIENT MAY BENEFIT FROM THROMBOLYTIC (IVT) OR EVT
May consider IVT therapy with any NIHSS score if below is met:
1. LSN <4.5 h ago, age ≥18 yo
2. CT brain negative for hemorrhage
3. Not on anticoagulation therapy
May consider EVT with NIHSS score ≥6 if below is met:
1. LSN <24 h, age ≥18 yo
2. CT brain negative for hemorrhage

CT, computed tomography; EVT, endovascular thrombectomy; IVT, intravascular thrombolysis; LSN, last seen normal; NIHSS, National Institutes of Health Stroke Scale.

## Results

### RATES OF TELESTROKE ACTIVATION AND AIS/TIA IN THE TELESTROKE-ELIGIBLE GROUP AMONG THOSE WHO SCREENED POSITIVE ON THE EMR REPORT

During this time period, 12,685 patients presented to these EDs. Of these patients, 252 (1.99%) screened positive and were pooled for analysis. Of the 252 patients, 12.7% (*n* = 32) met telestroke criteria. Of these potentials who met telestroke criteria, 43.75% (*n* = 14) received telestroke evaluation either by phone or audio-visual consult. A final diagnosis of AIS/TIA was made in 92.86% (*n* = 13) of these patients.

### RATES OF AIS AND TIA IN PATIENTS AMONG THE NONTELESTROKE-ELIGIBLE PATIENTS FROM THE EMR REPORT

**Fig. 1.**
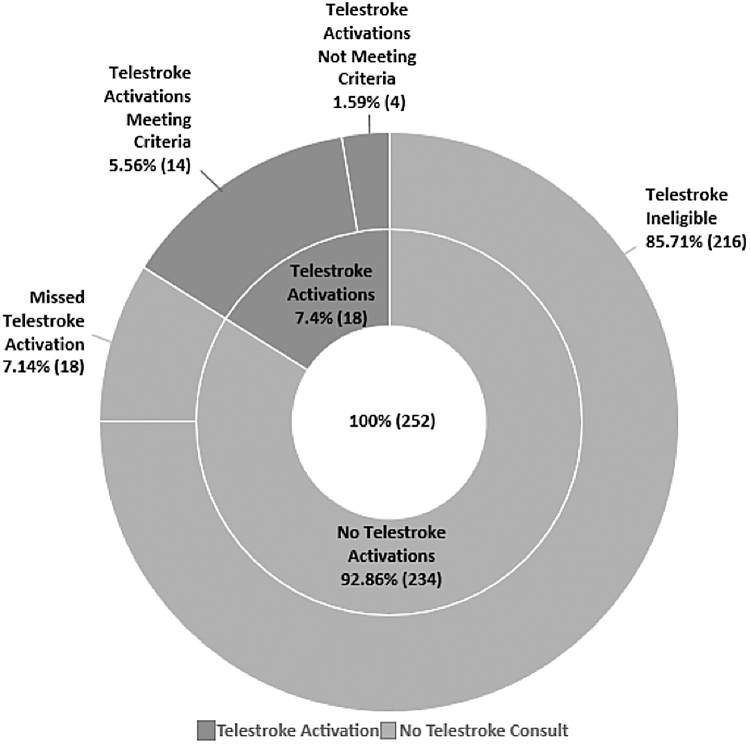
Telestroke activation versus no telestroke consult cases.

Of the 252 patients, 85.7% (*n* = 216) did not fulfill telestroke consultation criteria (shown in *Fig. 1*). A discharge diagnosis of AIS/TIA was made in 4.63% (*n* = 10) of these patients. Reasons that these AIS/TIA patients did not meet criteria were: being out of the IVT/EVT window (8) and having a NIHSS too low to meet EVT candidacy (2).

### FREQUENCY OF TELESTROKE ACTIVATIONS AMONG PATIENTS OUTSIDE ELIGIBILITY CRITERIA WHO SCREENED POSITIVE

Of all of the patients who underwent telestroke consultation (*N* = 18), 22.22% (*n* = 4) were activated despite failing to fulfill telestroke criteria. This represented 1.59% of the total patients pooled for analysis (*Fig. 1*). Of these patients, 75% (*n* = 3) ultimately received a diagnosis of AIS/TIA. Reasons that these specific patients did not meet telestroke activation criteria included being out of the IVT/EVT window (2) or being out of the IVT window with a NIHSS too low to meet EVT candidacy (*n* = 1).

### REASONS FOR NOT UTILIZING THE TELESTROKE SERVICE

Of the patients eligible for consult (*N* = 32), 56.25% (*n* = 18) did not undergo telestroke consultation. Upon chart review, reasons for not utilizing telestroke services included: symptoms resolving rapidly within the ED (*n* = 4), severity of presenting neurologic deficits deemed insufficient to warrant the risks of IVT administration by provider (*n* = 1), provider attributed symptoms to another medical process (*n* = 2), patient leaving against medical advice (AMA) (*n* = 1), provider comfortable managing AIS independent of the telestroke service (*n* = 4), lack of consideration for EVT therapy window (*n* = 3), recent stroke preventing use of IVT (*n* = 1), and unknown (*n* = 2) (shown in *Table 3*).

**Table 3.** Provider Reasons for Not Utilizing Telestroke Services

CHART-DOCUMENTED REASONS	NUMBER OF CASES BY FACILITY		
	CAH1	CAH2	CAH3
Symptoms judged too mild for IVT risk by provider	1	0	0
Lack of consideration for EVT therapy window	0	3	0
Patient leaving AMA	0	1	0
Provider comfortable with management without use of telestroke service	0	4	0
Symptoms resolving rapidly within the ED	1	1	2
Symptoms thought to be due to another medical process by provider	1	1	0
Unknown	2	0	0
Previous stroke precluding use of IVT	0	0	1

AMA, against medical advice; CAH1, critical access hospital 1; CAH2, critical access hospital 2; CAH3, critical access hospital 3; ED, emergency department; IVT, intravascular thrombolysis.

### ANALYSIS OF THE EMR REPORT

The EMR report pooled 252 potential cases of AIS/TIA out of a total of 12,685 ED visits during the 5-month period of this study. The report had a specificity of 98.22%, sensitivity of 58.06%, positive likelihood ratio of 32.68, negative likelihood ratio of 0.4269, and accuracy of 98.03%. Using the stroke prevalence of 4.4% for WV as reported by the WV Department of Health and Human Resources,^8^ the EMR report had a positive predictive value of 68.66% and a negative predictive value of 98.08%.

### PATIENTS WITH HOSPITAL DISCHARGE DIAGNOSIS OF AIS/TIA NOT IDENTIFIED ON THE EMR-DERIVED REPORT

Twelve thousand four hundred thirty-three of 12,685 (98.0%) patients seen were not picked up on the triage screen report. Of these, 33 (0.3%) were diagnosed with AIS/TIA by either the ED or admitting physician. Four of the thirty-three (12.1%) should have been included in the pooled report but were not for unknown reasons [Dizziness (3), Visual Loss (1)]. Another 9/33 (27.3%) patients presented with chief complaints concerning for stroke but fell outside our screening parameters with chief complaints as follows: Neurologic problem (*n* = 3), aphasia (*n* = 2), left sided weakness (*n* = 1), difficulty walking (*n* = 1), focal motor weakness (*n* = 1), vision loss (*n* = 1). Of the remaining 20/33 (60.1%) patients, the most common chief complaints were: altered mental status (AMS; *n* = 10) and weakness (*n* = 6).

## Discussion

In this observational study of telestroke utilization across three rural CAHs, the majority of telestroke-eligible (TSE) patients did not undergo telestroke consultation. In addition, the EMR-based report of stroke-related chief complaints proved to be a reasonably reliable tool to identify high risk AIS/TIA patients who are most likely to benefit from telestroke consultation.

Implementation and maintenance of telestroke networks require significant resources and continued investment from all network partners. Barriers that may present early on when establishing a new network include sustainability despite low patient volume and the ability to scale-up the network when faced with limited funding.^9,10^ Maintenance of an established telestroke network also presents challenges related to funding, staffing, technological difficulties, and the ability to standardize the triage process among all spoke sites.^9,10^ Despite these barriers, the benefits from a patient perspective are well-established.^11–13^ As with any resource-heavy tool, telestroke utilization should be wielded wisely. Likewise, once the investment is made, optimization must remain a top priority. Vital to these efforts is to understand the factors supporting the use of the network, as well as remaining barriers to overcome. Over half (56%) of the TSE patients in the current study did not generate a telestroke activation. It is this subset of patients who remain poorly characterized and represent our primary population of interest.

There were a number of immitigable reasons for patients not receiving consultation, such as patients leaving AMA, neurologic deficits thought to be due to another medical condition, and recent stroke preventing use of IVT. However, there were also cases where providers elected to manage and treat possible AIS independently rather than utilize the telestroke service. In contrast, cases were identified when providers failed to recognize the possibility of EVT interventions and thus falsely thought the patient to be telestroke ineligible. It is these latter two scenarios that are of interest for future interventions. Future efforts should be aimed at further understanding the barriers and reasons why providers choose not to utilize telestroke services, as well as to educate providers regarding EVT eligibility criteria, which may increase use of telestroke services. Potential variables to consider include critical access facility size, facility distance from hub site, and provider experience.

Moreover, the utility of a telestroke network may not solely be based in achieving the correct diagnosis and providing the most current treatment. Telestroke activation may facilitate coordinated transfers for patients not meeting strict criteria. Rural providers may also benefit from educational discussions and case review with hub site providers, and access to additional consultants may allow rural, single coverage providers to feel more supported in their jobs.

Among the nearly 13,000 ED visits studied, the EMR report flagged 252 (2.0%) as potentially high risk for stroke in evolution. Over the same period, 57/12,685 total TIA/AIS were identified through discharge diagnosis with a cumulative incidence of 0.45%. Although the proportion of strokes is somewhat smaller than incidence rates previously reported,^14^ the current study's data, to our knowledge, is the first to characterize rural CAHs' stroke presentations and telestroke utilization specifically. Therefore, these results provide crucial information on incidence of stroke-like events in rural populations. Rurality has previously been established as an independent predictor associated with lower acute stroke treatment rates and worse outcomes.^15,16^ AIS/TIA in general remains one of the most commonly missed diagnoses in the ED^17^ and represents an important opportunity to directly reduce morbidity with adequate secondary stroke prevention.^18,19^ Telestroke activation can effectively address these health care disparities.

Even though all originating sites were CAHs, intersite variations among telestroke activation among TSE ranged from 23% to 67% (Supplementary Data) and reasons (*Table 3*) for not utilizing the service were heterogeneous across sites. However, high specificity for true stroke cases was demonstrated with a high proportion of discharge diagnosis of AIS/TIA among activated patients across all three sites (100%, 60%, and 86%; Supplementary Data). Furthermore, the high rate of stroke diagnosis at discharge among the consulted patients who failed to fulfill the telestroke consult criteria demonstrates that the service is still being deployed among our target stroke population. Beyond the direct benefit of increasing acute stroke treatments, telestroke enhances care by streamlining follow-ups and serves as a robust source of peer-to-peer education between the telestrokologist and the bedside providers. Inferring definitive patterns or identifying fixed barriers based on the originating site, however, is limited by our small data set.

The EMR-derived report proved to be a viable tool to “rule-in” potential cases of AIS and TIA as evidenced by a specificity of 98.22%. Sensitivity of 58.06%, while reasonable, is not sensitive enough to rely on for clinical decision-making. Using total TIA/AIS discharges as a control revealed that 33 (0.3%) additional patients were identified. However, 4 (12.1%) recorded chief complaints that should have triggered the EMR tool but did not for unclear reasons. Another 9 (27.3%) presented with stroke-like chief complaints that were outside our screening parameters (neurologic problem, aphasia, left sided weakness, difficulty walking, focal motor weakness, vision loss). Future efforts should include streamlining the chief complaint field for providers to standardize the most accurate entry and expanding the possible presenting signs to include those missed here.

Future studies can investigate the report's potential as a tool for rural providers to identify patients that should strongly be considered for telestroke interventions. This report can also be used in data collection for other stroke research. To improve the sensitivity of this tool, the addition of other triage complaints strongly associated with stroke should be considered. Less specific triage complaints among patients ultimately diagnosed with TIA/AIS who were missed by our report included weakness and AMS. Including these latter chief complaints ballooned our potential stroke population to 1,500 possible encounters, illustrating that placing the priority on maximizing the tool's sensitivity occurs at too high a cost for the report to be used pragmatically. We therefore propose that while this small number of patients is an acceptable miss for the purposes of site/system planning and review, a triage tool cannot supplant the clinical evaluation.

Each telestroke case represents an opportunity to mitigate significant morbidity in resource-limited settings. Future efforts will focus on drilling down on why these were missed (i.e., individual factors and institutional factors) with the objective of maximizing telestroke utilization when appropriate.

## Limitations

This study has several limitations. In addition to those inherent to its retrospective design, review of three CAHs to target a stratified population produced an insufficient sample size for statistical measurements. Therefore, the studied population may be too small to be representative of all rural critical access sites. Rather, these findings should serve as primary data for further hypothesis generating efforts aimed at understanding how to optimize acute telemedicine services. Finally, the pooled sample size may inadequately represent the true number of missed stroke-like triage complaints due to incomplete chief complaint fields or presentations failing to appear on the prespecified list of potential chief complaints. This latter issue would be partially addressed with a selective expansion of designated chief complaints; however, increasing the sensitivity of the report to flag all possible chief complaints associated with patients who are subsequently discharged with AIS/TIA would negate the usefulness of the tool.

## Conclusion

In this retrospective review among three CAHs, we found that: (1) the majority (56%) of TSE patients did not undergo telestroke consultation and (2) EMR report based on chief complaint is a reasonable method to identify those patients most likely to benefit from telestroke consultation. Future studies are critical to further understand barriers and reasons why providers choose not to use telestroke services.

## Supplementary Material

Supplemental data

Supplemental data
