# Supplementary material for: Analysis of Telestroke Usage in Rural Critical Access Emergency Departments
Source: Telemed J E Health. 2023 Dec 8;29(12):1828–33. doi: 10.1089/tmj.2022.0408 (PMC10714255; doi:10.1089/tmj.2022.0408)
Supplement: Supplemental data [file Suppl_Data.doc]

**Supplemental data**

*Hospital-Specific Data*

Of the 56 total potentials at JAX, 17.86% (n=10) met telestroke criteria. Of these potentials who met criteria, 50% (n=5) received telestroke consultation. One activation did not meet telestroke criteria however, 100% (n=6) of their total activations were diagnosed with AIS/TIA.

Of the 140 total potentials at SMR, 9.29% (n=13) met telestroke criteria. Of these potentials who met criteria, 23.08% (n=3) received telestroke consultation. Two activations did not meet telestroke criteria. 60% (n=3) of their total activations were diagnosed with AIS/TIA.

Of the 56 total potentials at BRX, 16.07% (n=9) met telestroke criteria. Of these potentials who met criteria, 66.67% (n=6) received telestroke consultation. One activation did not meet telestroke criteria. 100% (n=7) of their total activations were diagnosed with AIS/TIA.

Quantifying interfacility differences was hard to ascertain due to the low power when comparing these facilities individually. However, when looking at generalized trends, the following was found. Overall, BRX had the highest rates of telestroke utilization (66.67%) while SMR had the lowest telestroke utilization rate (23.08%). However, BRX and JAX had the highest specificities (33.33%) in selecting AIS/TIA cases for telestroke services while BRX had the highest sensitivity (85.71%) in selecting AIS/TIA cases for telestroke services. All three of these facilities are critical access hospitals serving rural populations. Refer to figures 2 and 3 for hospital-specific comparisons between these three facilities.
