# Supplementary figures and images for: Analysis of Telestroke Usage in Rural Critical Access Emergency Departments
Source: Telemed J E Health. 2023 Dec 8;29(12):1828–33. doi: 10.1089/tmj.2022.0408 (PMC10714255; doi:10.1089/tmj.2022.0408)

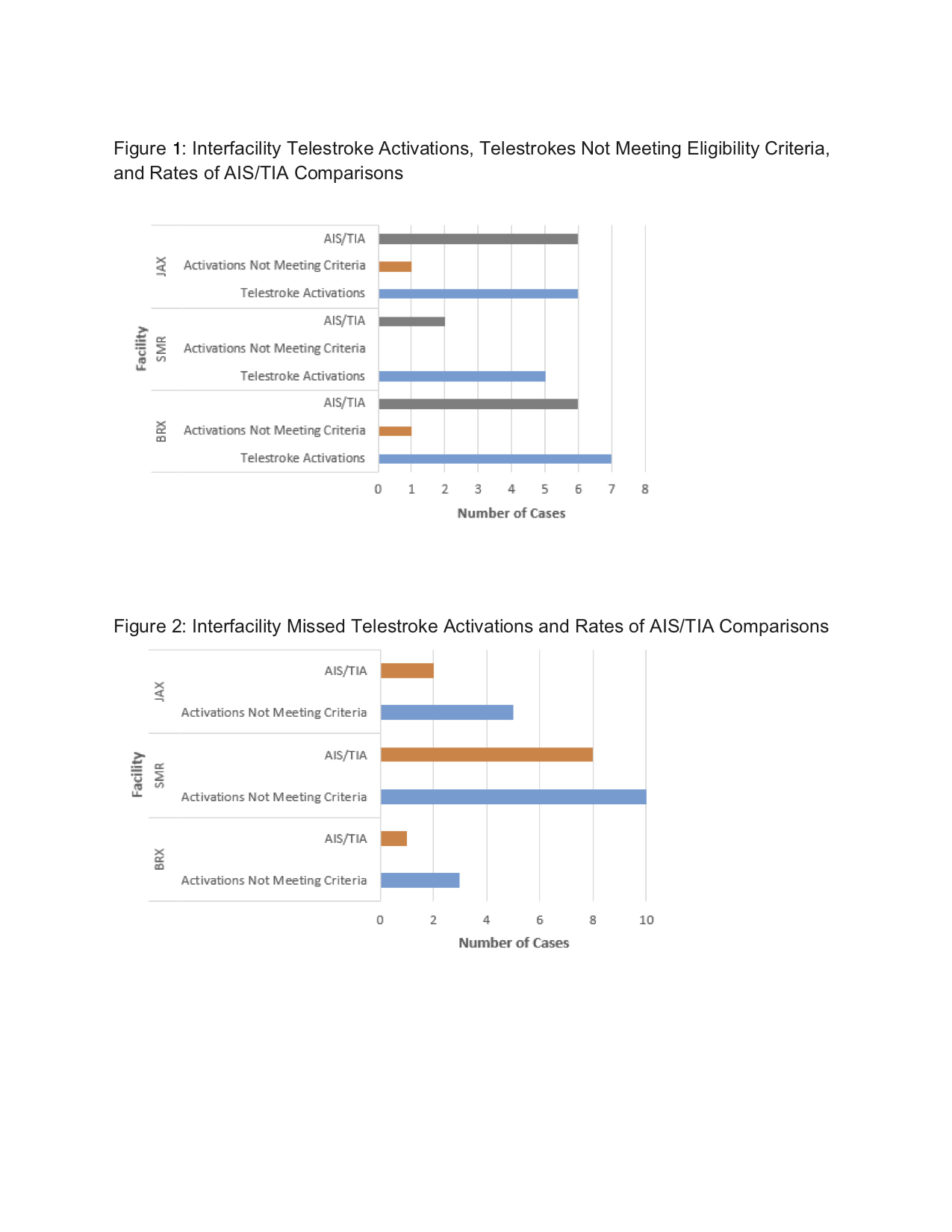

Supplement: Supplemental data [file Suppl_FigS1-S2.jpg]
